# Cyan fluorescent proteins derived from mNeonGreen

**DOI:** 10.1093/protein/gzac004

**Published:** 2022-05-09

**Authors:** Landon Zarowny, Damien Clavel, Ryan Johannson, Kévin Duarte, Hadrien Depernet, Jérôme Dupuy, Heather Baker, Alex Brown, Antoine Royant, Robert E Campbell

**Affiliations:** 1 Department of Chemistry, University of Alberta, Edmonton T6G 2G2, Canada; 2 Univ. Grenoble Alpes, CNRS, CEA, Institut de Biologie Structurale (IBS), 38000 Grenoble, France; 3 Structural Biology Group, European Synchrotron Radiation Facility, 38043 Grenoble, France; 4 Department of Chemistry, University of Tokyo, Tokyo 113-0033, Japan

**Keywords:** crystal structure, fluorescence microscopy, fluorescent protein, protein engineering, tryptophan-derived chromophore

## Abstract

mNeonGreen, an engineered green fluorescent protein (GFP) derived from lancelet, is one of the most brightly fluorescent homologs of *Aequorea victoria* jellyfish GFP (avGFP) yet reported. In this work, we investigated whether this bright fluorescence might be retained in homologs of mNeonGreen with modified chromophore structures and altered fluorescent hues. We found mNeonGreen to be generally less tolerant than avGFP to chromophore modification by substitution of the key chromophore-forming tyrosine residue with other aromatic amino acids. However, we were ultimately successful in creating a variant, designated as NeonCyan1, with a tryptophan-derived cyan fluorescent protein (CFP)-type chromophore, and two additional mutants with distinct spectral hues. Structural, computational, and photophysical characterization of NeonCyan1 and its variants provided insight into the factors that control the fluorescence emission color. Though not recommended as replacements for contemporary CFP variants, we demonstrate that NeonCyan1 variants are potentially suitable for live cell imaging applications.

## Introduction

**Fig. 1:**
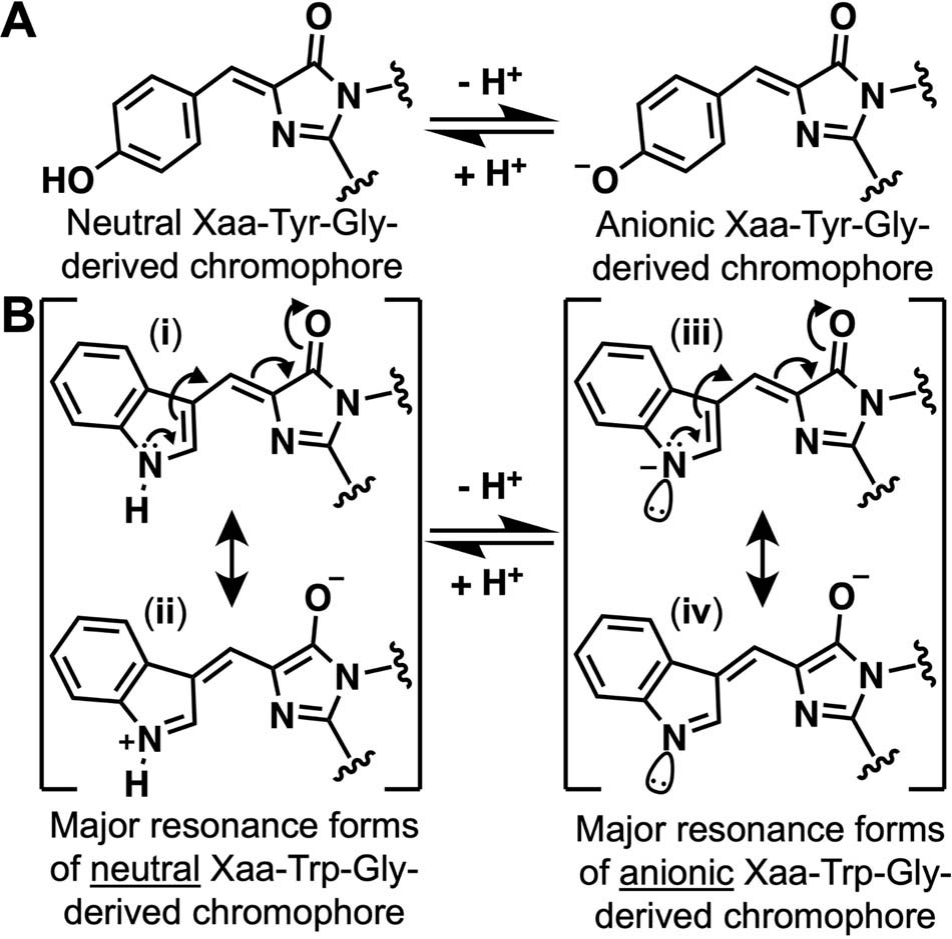
Chromophore structures of engineered GFP and CFP variants. (**A**) Wild-type avGFP and mNeonGreen chromophores are formed from the Xaa-Tyr-Gly tripeptide. The first amino acid of the tripeptide (Xaa) is serine in avGFP (Prasher *et al.* 1992), threonine in enhanced GFP (EGFP) (Cormack *et al.* 1996), and glycine in mNeonGreen (Shaner *et al.* 2013). (**B**) CFP and NeonCyan1 chromophores are formed from the Xaa-Trp-Gly tripeptide. The first amino acid of the tripeptide (Xaa) is threonine in avGFP-derived enhanced CFP (ECFP) (Tsien 1998). An anionic form of the Xaa-Trp-Gly has been proposed for certain CFP variants that exhibit red-shifted fluorescence emission (Pletnev *et al.* 2015; Sarkisyan *et al.* 2012, 2015a). Literature representations of the anionic form have placed the negative charge on the indole nitrogen (**iii**), but the negative charge is almost certainly distributed between this atom and the more electronegative oxygen atom in the 4-imidazolone moiety (**iv**).

Cyan fluorescent proteins (CFPs) are an important class of fluorescent protein (FP) due to their utility in multicolor imaging applications and as donors in Förster resonance energy transfer pairs (Tsien 1998). CFPs (Cubitt *et al.* 1995; Heim *et al.* 1994), blue FPs (BFPs) (Ai *et al.* 2007), and so-called ultramarine FPs (UMFPs) (Tomosugi *et al.* 2009) are a unique subset within the FP palette because they are defined by mutation of the key chromophore-forming tyrosine (Fig. 1A) to one of the other aromatic amino acids, such as tryptophan for the CFP class (Fig. 1B). Similar modifications have also been reported for the further extended chromophores of red FPs (Ai *et al.* 2007; Battad *et al.* 2012; Pletneva *et al.* 2015; Sarkisyan *et al.* 2015b; Shaner *et al.* 2004).

Since the first report of a mutagenized FP with an Xaa-Trp-Gly chromophore (Heim *et al.* 1994), the CFP lineage of FPs has been steadily and substantially improved through the use of protein engineering. Two of the brightest and most recent variants are mCerulean3 (Markwardt *et al.* 2011) with a quantum yield (Φ) of 0.87 and an extinction coefficient (ε) of 31 000 mM^−1^ cm^−1^, and mTurquoise2 (Goedhart *et al.* 2012) with Φ = 0.93 and ε = 30 000 mM^−1^ cm^−1^ (Table I). Introduction of basic amino acids adjacent to the chromophore of mCerulean (Rizzo and Piston 2005) led to the discovery of green fluorescent protein (GFP) variants, such as NowGFP (Table I), that are proposed to harbor the anionic state of the tryptophan-derived chromophore (Pletnev *et al.* 2015; Sarkisyan *et al.* 2012, 2015a) (Fig. 1B).

**Table I:** Fluorescence properties of FPs discussed in this work

Protein	λ_ex_ (nm)	λ_ex_ (nm)	ε at 1° peak (mM^−1^ cm^−1^)	Φ	Brightness^a^	Apparent p*K*_a_
mNeonGreen^b^	506	517	116	0.80	93	5.7
NeonCyan1	430 (1°)^a^ 454 (2°)	480 (1°) 498 (2°)	29	0.29	8.4	6.4
NeonCyan1-T207M	410 (1°) 424 (2°)	460 (2°) 485 (1°)	29	0.18	5.2	6.5
NeonCyan1-T207D	478	504	42	0.27	11	5.4, 8.0
NeonCyan1-truncated	430 (1°) 454 (2°)	480 (1°) 498 (2°)	28	0.27	7.6	6.7
mCerulean3^c^	433 (1°) 450 (2°)	475 (1°) 496 (2°)	31	0.87	27	3.2
mTurquoise2^d^	434 (1°) 452 (2°)	474 (1°) 502 (2°)	30	0.93	28	3.1
NowGFP^e^	494	502	57	0.76	43	6.2

^a^For both excitation and emission, 1° indicates the peak maximum and 2° indicates a second local peak maximum in the spectral profile.

^b^Product of ε at 1° peak in mM^−1^ cm^−1^ and Φ.

^c^Values from Shaner *et al.* (2013).

^d^Values, other than ε, are from Markwardt *et al.* (2011).

^e^Values from Goedhart *et al.* (2012).

^f^Values from Sarkisyan *et al.* (2015a).

The large majority of FPs used in microscopy are derived from wild-type FPs cloned from animals in the phylum cnidaria, which includes jellyfish, sea anemones, and corals (Alieva *et al.* 2008). A much smaller number of FPs have been cloned from bilaterian animals including copepods in phylum arthropoda (Shagin *et al.* 2004), and lancelets in phylum chordata (Baumann *et al.* 2008; Deheyn *et al.* 2007). Further engineering of one particular lancelet-derived FP, LanYFP, yielded one of the brightest monomeric GFPs yet reported, mNeonGreen (Table I) (Shaner *et al.* 2013). Owing to its exceptional brightness and performance for applications in fluorescence microscopy, mNeonGreen has become widely accepted as a brighter alternative to avGFP variants. mNeonGreen has also proven versatile enough to be used for construction of high-performance genetically encoded Ca^2+^ biosensors (Subach *et al.* 2020; Zarowny *et al.* 2020).

Following the precedent set by the original development of the BFP, CFP, and UMFP derivatives of avGFP (Cubitt *et al.* 1995; Heim *et al.* 1994), we have attempted to make analogous modifications to the Gly-Tyr-Gly-derived chromophore of mNeonGreen. This effort ultimately led to the development of three blue shifted variants, with Gly-Trp-Gly-derived chromophores, which we have named NeonCyan1, NeonCyan1-T207D, and NeonCyan1-T207M. Here we describe the development, characterization, and structures of these variants, and briefly explore their potential to be applied for applications in fluorescence microscopy.

## Materials and Methods

### Sources of materials used

Synthetic DNA oligonucleotides and gBlocks were purchased from Integrated DNA Technologies (IDT) (Supplementary Table I). Plastic consumables, restriction endonucleases, *Taq* polymerase, Phusion polymerase, T4 DNA ligase, deoxynucleotides, DH10B *Escherichia coli.*, pBAD/His B plasmid, pcDNA3.1(+) plasmid, Bacterial Protein Extraction Reagent (B-PER), Penicillin–Streptomycin, Fetal Bovine Serum (FBS), TurboFect, and GeneJET gel and plasmid purification kits were purchased from ThermoFisher. Agarose, MnCl_2_·4H_2_O_,_ D-glucose, ampicillin, L-arabinose, Hank’s balanced salt solution (HBSS), Dulbecco’s Modified Eagle Medium (DMEM), TryplE Express, and LB Lennox media were purchased from Fisher Scientific. Nickel nitrilotriacetic acid (NTA) immobilized metal affinity chromatography protein purification beads were purchased from G-BioSciences. Ethidium bromide and polymerase chain reaction (PCR) instruments (T100 Thermal Cycler) were purchased from BioRad. Gibson Assembly reagent was purchased from New England Biolabs. QuikChange mutagenesis kits were purchased from Agilent Technologies. Nunc MicroWell 96-Well Optical-Bottom Plates (catalog #265301) were purchased from ThermoFisher. Molecular weight cutoff filters were purchased from Millipore-Sigma. Sequencing was performed by the Molecular Biology Services Unit (MBSU) at the University of Alberta.

### PCR and mutagenesis

All PCR reactions were performed according to the polymerase manufacturer’s recommendations. PCR reactions utilizing Phusion polymerase were performed in the supplied buffer with the concentrations of DNA and oligos suggested by the manufacturer. Cycling conditions were 30 cycles of 98°C for 10 s, 63°C for 20 s, 72°C for 30 s, and 72°C for 60 s for final extension. PCR products were run on 1% agarose gel in TAE buffer, excised after viewing on a UV transilluminator, and recovered with the ThermoFisher gel preparation kit according to the manufacturer’s recommendations.

Site-directed mutagenesis was performed using the QuikChange Lightning site-directed mutagenesis kit (Agilent) according to the manufacturer’s instructions. Electrocompetent *E. coli* DH10B (50 μl) was transformed with the reaction product (2 μl) using an electroporation cuvette (0.2 cm, Bio-Rad) and a Bio-Rad micropulser (*E. coli* 2 setting). The DH10B cells were immediately resuspended in room temperature LB (1 ml) and plated at a density of ~250 colonies per plate. Oligos for site-directed mutagenesis reactions were designed using the Agilent QuikChange Primer Design online tool (www.agilent.com/store/primerDesignProgram.jsp) and synthesized by IDT. To create libraries by site-directed saturation mutagenesis we used these same conditions but incorporated NNK codons (N = A, G, C, or T and K = G or T) at the positions of residues targeted for mutagenesis.

To create libraries with randomly generated mutations, error-prone (EP)-PCR (Cadwell and Joyce 1992) was performed using *Taq* polymerase with 0.1 mM MnCl_2_ and a skewed dNTP ratio (0.2 mM ATP, 1 mM CTP, 0.2 mM GTP, 1 mM TTP), resulting in ~1 nucleotide mutation per 800 base pairs. Cycling conditions were 35 rounds of 95°C for 20 s, 55°C for 30 s, 72°C for 60 s, and 5 min at 72°C for final extension.

Gene libraries were inserted into the pBAD/His B vector by ligation of a XhoI and HindIII digested PCR product. Briefly, an EP-PCR reaction (50 μl) was performed as above, the product run in a 2% agarose gel containing ethidium bromide, the band excised, and the DNA product purified using the GeneJET Gel Extraction Kit from ThermoFisher. The PCR product (3 μg) was digested for 1 h at 37°C with two units each of XhoI and HindIII. The digested DNA was purified again using the GeneJET Gel Extraction Kit. The digested product was used in a 20 μl T4 ligase reaction containing 100 ng of XhoI and HindIII digested pBAD/His B plasmid with a 7:1 ratio of insert to vector. The ligation reaction was incubated for 1 h at room temperature before being used to transform *E. coli* DH10B as described above. Plasmid DNA was recovered from an *E. coli* overnight culture (4 ml) using the GeneJET Plasmid Miniprep Kit (ThermoFisher) according to the manufacturer’s recommendations.

### Directed evolution

The NeonCyan gene libraries in the pBAD/His B vector were used to transform DH10B *E. coli* bacteria which was plated onto 1.5% agar plates containing 100 μg/ml ampicillin and 0.02% L-arabinose and grown overnight (12–18 h) at 37°C. Images acquired using a fluorescent colony screening system (Ai *et al.* 2014) were analyzed with Image-Pro 6 software (Media Cybernetics) to identify the brightest fluorescent colonies which are then picked and grown in LB Lennox growth medium (4 ml) containing 0.02% L-arabinose and 100 μg/ml ampicillin. The overnight culture was centrifuged and the bacterial pellet was extracted with B-PER. The FP-containing lysate was dispensed into 96-well plates and the fluorescence excitation and emission spectra were measured on a Tecan Safire^2^ plate reader.

To evaluate the relative brightness of variants in a library, the integrated fluorescent emission intensity was normalized to the absorbance at 280 nm for each variant. Plasmid DNA from variants with the highest normalized fluorescence intensity were purified using the GeneJET Plasmid Miniprep Kit and used as the template for subsequent library generation by EP-PCR. Every few rounds during the evolution campaign, a selection of variants were purified and characterized in terms of quantum yield and extinction coefficient to ensure that the directed evolution was indeed leading to the discovery of brighter variants.

### Protein expression and purification

To purify NeonCyan variants for routine characterization, the pBAD/His B plasmid containing the gene encoding the desired variant was used to transform electrocompetent DH10B *E. coli* which were then plated on LB agar plates supplemented with ampicillin (400 μg/ml). Following overnight incubation at 37°C, a single colony was picked and used to inoculate 500 ml of LB liquid media with ampicillin (200 μg/ml) and 0.02% L-arabinose. Bacterial cultures were grown for 30 h at 37°C followed by 24 h at 30°C, with shaking at 250 rpm. Cells were harvested by cooling on ice to 4°C then centrifugation (6000 × g for 6 min), re-suspended in 30 ml of ice-cold TBS buffer (pH 7.2), and lysed by sonication (QSonica Q700, amplitude 50, 1 s on, 2 s off, 2 min sonication time). All subsequent purification procedures were performed on ice. The lysate was clarified of cell debris by centrifugation for 1 h at 21 000 × g, filtered through a Kim-wipe into a 50 ml conical tube, and incubated for 3 h with Ni-NTA resin. The resin was washed with 100 ml of 20 mM imidazole TBS wash buffer and eluted with 250 mM imidazole TBS elution buffer. Purified protein was buffer exchanged into TBS using a 10 000 Da molecular weight cutoff filter (Millipore-Sigma) through three successive washes. Absorption spectra were recorded on a Beckman-Coulter DU-800 UV–visible spectrophotometer and fluorescence spectra recorded on a Tecan Safire^2^ plate reader.

The overexpression and purification of NeonCyan0.95 for crystallization was performed as previously described (Lelimousin *et al.* 2009). A colony of *E. coli* DH10B transformed with the pBAD/His B plasmid containing the gene encoding NeonCyan0.95 was used to inoculate 1 L of LB medium which was grown at 37°C until OD600 reached 1.25. Protein expression was then induced overnight at 17°C with the addition of 10 ml of L-arabinose (20% w/v). Cells were centrifuged at 4000 × g during 20 min (Avanti J-26 XP, Beckman Coulter) and pellets were resuspended, prior to flash-freezing at −80°C, with 20 ml of lysis buffer made of 50 mM Tris pH 8.0, 300 mM NaCl, 10 mM imidazole, 0.25 mg/ml lysozyme, 400 μg/ml DNAse I and anti-protease cocktail (Complete EDTA-free, Roche). Sonication was carried out on an ice bath with five cycles of 10 s at 50% maximum power. Cell debris underwent centrifugation at 17 500 × g during 30 min at 4°C. The tagged protein was separated from the clarified lysate using a nickel affinity column (HisTrap QFF 5 ml, GE Healthcare) followed by size exclusion chromatography (Superdex 75 10/300 GL, GE Healthcare). This procedure produced a 20 mg/ml protein solution with a yield of 15 mg/L of medium. Prior to crystallization, the purified proteins were submitted to partial tryptic digestion for 1 h at room temperature at a 1:10 trypsin:protein ratio.

### X-ray crystallographic methods

Crystallization screening was performed on NeonCyan0.95 at the at the HTX Lab (EMBL Grenoble, Fr) using 288 distinct conditions (JCSG-plus from Molecular Dimensions, and Wizard Classic I & II from Rigaku). The initial hit condition was reproduced and refined to 0.1 M HEPES pH 7.5, 18% PEG8000 (w/v) using the hanging drop vapor diffusion method with a drop size of 2 μl (ratio protein/precipitant 1:1) at 293 K and a protein concentration of 20 mg/ml. Needle-shaped crystals grew within a week.

Prior to automated data collection at beamline MASSIF-1 (Bowler *et al.* 2016) of the European Synchrotron ESRF, a NeonCyan0.95 crystal was progressively cryoprotected with a solution of mother liquor supplemented with 20% (v/v) glycerol. Data were collected at 100 K, integrated and reduced in space group P1 using autoPROC (Kabsch 2010) taking data anisotropy into account (Tickle *et al.* 2018) (Supplementary Table II). The structure of NeonCyan0.95 at physiological pH was solved by the molecular replacement method with the program Phaser (McCoy *et al.* 2007) using the structure of mNeonGreen at near physiological pH (PDB ID: 5LTR) as a search model (Clavel *et al.* 2016). The asymmetric unit contains four molecules of NeonCyan0.95. Model rebuilding was carried out in the visualization software Coot (Emsley *et al.* 2010) and the structure was refined with Refmac5 (Murshudov *et al.* 2011). Structure refinement statistics are provided in Supplementary Table II. UCSF Chimera was used for modeling and representations of NeonCyan0.95 (Pettersen *et al.* 2004).

Crystals of NeonCyan0.95 at acidic pH were obtained with 0.1 M sodium acetate trihydrate, 24% PEG4000 (w/v), and 0.2 M ammonium sulfate, using the hanging drop vapor diffusion method with a drop size of 4 μl (ratio protein/precipitant 3:1) at 293 K and a protein concentration of 20 mg/ml. The pH of the buffer solution was originally adjusted to pH 8, however since the p*K*_a_ of acetate is 4.6, the actual pH of the crystallization condition was later estimated to be closer to ~ 5.6 (i.e., the highest pH value of the buffering range). Data from a crystal cryoprotected with 20% glycerol (v/v) were collected at 100 K on beamline BL13-XALOC of the Spanish synchrotron ALBA (Juanhuix *et al.* 2014) and reduced in the P1 space group with the XDS program suite (Kabsch 2010), with eight molecules in the asymmetric unit. Model building and refinement was performed as described above.

Crystals of NeonCyan1-T207D were obtained with 0.1 M HEPES pH 6.5 and 22% PEG8000 (w/v) using the hanging drop vapor diffusion method with a drop size of 3 μl (ratio protein/precipitant 2:1) at 293 K and a protein concentration of 20 mg/ml. Data from a crystal cryoprotected with 20% glycerol (v/v) were collected at 100 K on beamline BL13-XALOC and reduced in the P1 space group with four molecules in the asymmetric unit. Model building and refinement was performed as described above.

All three structures have been deposited in the Protein Data Bank, under accession codes 7Z7O for the structure of NeonCyan0.95 at pH 7.5, 7Z7P for the structure of NeonCyan0.95 at pH 5.6 and 7Z7Q for the structure of NeonCyan1-T207D.

### Spectral and biophysical characterization

Extinction coefficients were determined using the alkaline denaturation method (Cranfill *et al.* 2016). Briefly, the absorption spectrum of each protein was determined in both TBS buffer and 2 M NaOH. Absorbance of the denatured FP at 460 nm was divided by the previously determined extinction coefficient of 46 000 M^−1^ cm^−1^ to give the concentration of protein (Sarkisyan *et al.* 2012). The extinction coefficient was then determined by dividing the TBS sample absorbance maximum by the protein concentration. We observed that the denaturation of NeonCyan variants is effectively immediate but the denaturation of mCerulean3 takes at least 5 min to reach a maximum. Upon treatment with 2 M NaOH, a minimal change in NeonCyan absorbance was observed over a 10 min period.

Quantum yields were determined by first adjusting the concentration of each protein by dilution with TBS to reach an absorbance of 0.6 to 1.0. A dilution series was then prepared with absorbances of 0.01, 0.02, 0.03, 0.04, and 0.05 for NeonCyan variants and mCerulean3. Recording and integration of the fluorescent emission peaks provided a total fluorescent emission value which was plotted against the absorbance to provide a slope. The quantum yields of NeonCyan variants were determined using the published QY value (Cranfill *et al.* 2016) according to the equation

$${\Phi}_{\mathrm{protein}}={\Phi}_{\mathrm{standard}}\times \left({\mathrm{S}}_{\mathrm{protein}}/{\mathrm{S}}_{\mathrm{standard}}\right)$$

where S is the slope of integrated intensity of fluorescent emission from a constant excitation wavelength.

Absorbance spectra of NeonCyan0.95, NeonCyan1-T207M, NeonCyan1-T207D, and mNeonGreen were measured using a BeckmanCoulter DU800 UV–Vis spectrophotometer. Purified protein was diluted to 0.1 mg/ml in TBS buffer pH 7.4 or TBS buffer pH 7.4 with 10 mM K_2_SO_4_ and the spectra collected immediately. Low pH citrate buffered solutions were used to obtain spectra at 0.1 mg/ml for variants with and without K_2_SO_4_.

The apparent p*K*_a_s of NeonCyan variants were determined using a buffer series created following Carmody’s (1961) method and the protocol of Cranfill *et al.* (2016). Briefly, 2 μg of purified protein was added to 100 μl of each respective pH buffer in a black-walled 96-well microplate. Each variant was measured in triplicate and background corrected before normalization of the data. The p*K*_a_ value was determined with GraphPad Prism 7 software using a four-parameter variable-slope fit.

Size-exclusion fast protein liquid chromatography (FPLC) was performed on a Superdex 75 10/300 GL (GE Healthcare), fed by an AKTA P-900 pump (GE Amersham). Chromatography was performed using 2 μg of each protein sample and detected with a UV-900 monitor (GE Amersham). Absorbance signals were detected at 430, 430, 505, and 555 nm for NeonCyan1, NeonCyan1-truncated, mNeonGreen, and tdTomato, respectively.

### Computational methods

A model of the isolated chromophore was built using the experimentally determined structure of the mNeonGreen chromophore (PDB ID: 5LTR) (Clavel *et al.* 2016). Models for the anionic and neutral forms were created by adding or removing hydrogen atoms as required. Starting from the original geometry, optimization was performed using the ωB97XD functional (Chai and Head-Gordon 2008) and the cc-pVDZ basis set (Dunning 1989) in Gaussian (Frisch *et al.* 2016). The geometries were confirmed as minima through vibrational frequency analysis. The optimization was completed both in gas-phase and using an integral equation formalism polarizable continuum model (IEF-PCM) for solvation (Cancès *et al.* 1997; Scalmani and Frisch 2010; Tomasi *et al.* 2005) to simulate the isolated chromophore in water. Using the optimized geometries of both the anionic and neutral chromophores, time-dependent density functional theory (TD-DFT) computations were completed in both the gas-phase and water using IEF-PCM (Cossi and Barone 2000, 2001) using the same functional and basis set to determine the first excited state energies and oscillator strengths.

### Mammalian cell expression and imaging

PCR amplified genes encoding NeonCyan1 variants were digested with Xho1 and HindIII and ligated into a modified and suitably digested pcDNA3.1 (ThermoFisher) vector to create pcDNA-NeonCyan1. These pcDNA-NeonCyan1 vectors were amplified with primers that produced a linear product suitable for Gibson Assembly. These linear vectors were combined with a synthetic human codon optimized gBlock fragment from IDT (F-tractin gBlock) using Gibson Assembly. The resulting product was used to transform *E. coli* DH10B, single colonies were picked, and plasmid DNA was isolated and sequenced to identify insertions with no frameshifts or mutations.

HeLa cells cultured in DMEM with 10% FBS supplemented with penicillin-G potassium salt (50 units/ml) and streptomycin sulfate (50 μg/ml) were plated on collagen coated 35 mm glass bottom dishes (Matsumami) and transfected with 1 μg of plasmid DNA using 2 μl of TurboFect when cells reached roughly 60% confluency. After overnight incubation at 37°C with 5% CO_2_, adherent cells were washed twice with warm HBSS immediately before imaging. Cell imaging was performed on a Zeiss 200 M wide-field microscope equipped with an OrcaFlash 4.0—C13440 (Hamamatsu) camera, an MS-2000 automated stage (Applied Scientific Instrumentation), and a 60× oil objective lens (N.A. 1.4). Exposure times for NeonCyan1 and NeonCyan-T207D were 25 ms and for NeonCyan1-T207M was 100 ms using the same gain and illumination intensity. Semrock filters specific for each NeonCyan1 variant were used as follows: NeonCyan1-T207M excitation 387/11 nm and emission 460/40 nm; NeonCyan1 excitation 436/20 and emission 483/32 nm; NeonCyan1-T207D excitation 470/40 nm and emission 525/50 nm. Images were acquired using MetaMorph 7.8.0.0 software and analyzed and pseudo-colored with ImageJ2 (Rueden *et al.* 2017; Schneider *et al.* 2012).

## Results

### Development of NeonCyan1

In an effort to develop variants of mNeonGreen with alternative chromophore structures, we created three gene libraries in which the second residue of the chromophore-forming tripeptide (Tyr69) was mutated to phenylalanine (Tyr69Phe), histidine (Tyr69His), or tryptophan (Tyr69Trp), and the first residue of the chromophore (Gly68) was mutated to all 20 common amino acids. No fluorescent variants were found in either the Tyr69Phe or Tyr69His libraries. A dimly fluorescent variant was discovered in the Tyr69Trp library that retained a glycine at position 68. This variant, designated NeonCyan0.1, served as the starting point for an extensive process of directed evolution for improved brightness.

**Fig. 2:**
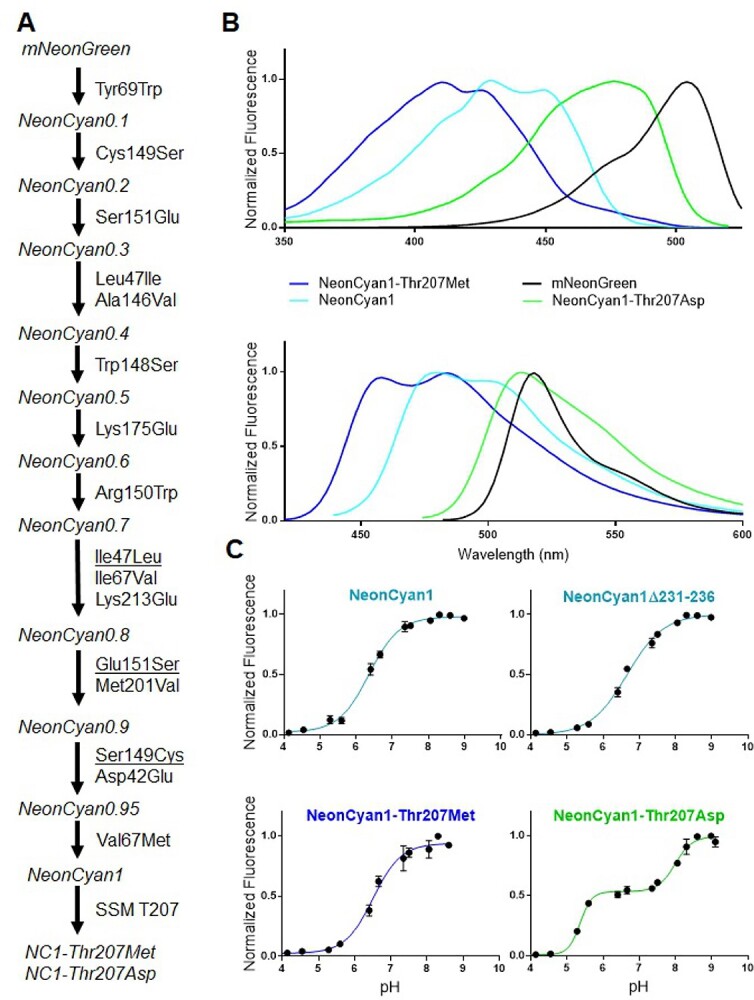
Lineage, spectral profile, and pH titration of NeonCyan1 variants. (**A**) Lineage of NeonCyan1, mutations that were reverted during the directed evolution campaign are underlined. SSM: Site-saturation mutagenesis. (**B**) Excitation spectra (upper panel), and emission spectra (lower panel), of NeonCyan1, NeonCyan1-T207M, NeonCyan1-T207D, and mNeonGreen. (**C**) pH titrations of NeonCyan1 variants.

To perform directed evolution, gene libraries were created through a combination of EP-PCR (Wilson and Keefe 2000) and site-saturation mutagenesis (Jones and Howard 1990), and screened in the context of colonies of *E. coli*. Sites of beneficial mutations discovered in libraries generated by EP-PCR were typically further investigated by randomization to all 20 common amino acids using site-saturation mutagenesis. When multiple improved variants were discovered in a round of screening from a library produced by EP-PCR, the corresponding plasmids would be mixed and mutations shuffled using staggered extension PCR (Zhao *et al.* 1998). After 10 rounds of evolution (Fig. 2A), it was apparent the protein had plateaued in terms of brightness improvements and we decided to halt the process of directed evolution.

The final variant, designated as NeonCyan1, has nine mutations relative to mNeonGreen: Asp42Glu, Val67Met, Tyr69Trp, Ala146Val, Trp148Ser, Arg150Trp, Lys175Glu, Met201Val, and Lys213Glu (Supplementary Fig. 1). NeonCyan1 has the ‘double humped’ excitation and emission spectra (Fig. 2B) that are characteristic of FPs with Trp-derived chromophores in the neutral state, such as mTurquoise2 and mCerulean3 (Table I). A late-stage variant, designated as NeonCyan0.95 (with Val67Ile rather than Val67Met), was used for structure determination by X-ray crystallography.

### X-ray crystal structure of NeonCyan0.95 at physiological pH

**Fig. 3:**
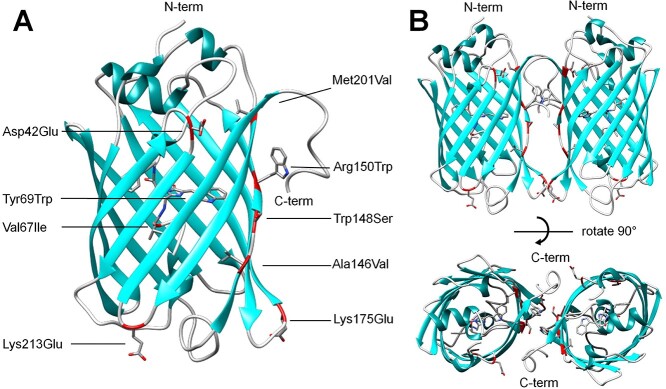
NeonCyan0.95 mutations and putative dimer structure. (**A**) The location of mutations in NeonCyan0.95 is highlighted using a red ribbon backbone representation and side chains shown as stick bonds. (**B**) Two views of the putative A-C dimer of NeonCyan0.95 with mutations highlighted as in (**A**).

To determine the atomic structure of NeonCyan0.95 at physiological pH, the protein was crystallized at pH 7.5 (Supplementary Fig. 2A), X-ray diffraction data were collected to 1.64 Å resolution, and the crystal structure was solved by molecular replacement using a previously reported structure of mNeonGreen (Clavel *et al.* 2016). Inspection of the NeonCyan0.95 crystal structure reveals the locations and interactions of all mutations introduced during directed evolution. Notably, six of the nine mutations (Fig. 3A) appear to be localized in a new dimer interface between monomers A and C of the asymmetric unit (Fig. 3B). The asymmetric unit contains four molecules of NeonCyan0.95 (Supplementary Fig. 2B).

Calculation of the interaction surfaces between the various monomers (779 Å^2^ between monomers A and C, 439 Å^2^ between A and B, 312 Å^2^ between A and D, 272 Å^2^ between B and D, 272 Å^2^ between B and D, and 0 Å^2^ between B and C) strongly suggests that the symmetrical dimer formed by monomers A and C is physiologically relevant, while other contacts are likely due to crystal packing. The C-terminal MDELYK sequence folds into a helical conformation and contributes to the interaction interface. To determine if the dimeric structure observed in the crystal structure was retained in the fully soluble state, we subjected NeonCyan1 to size-exclusion FPLC. NeonCyan1 eluted between the peaks for monomer and dimer standards, suggesting that it exists in a monomer-dimer equilibrium (Supplementary Fig. 3). Based on the crystal structure, we speculated that interactions of the structured C-terminal ‘MDELYK’ tail of NeonCyan1 was contributing to the stability of the dimeric species. Truncation of the C-terminal ‘MDELYK’ tail of NeonCyan1 (NeonCyan1-truncated) had no substantial effect on the photophysical properties of the protein (Table I) but did cause the protein to behave more like the mNeonGreen monomeric standard when analyzed by size-exclusion chromatography (Supplementary Fig. 3).

**Fig. 4:**
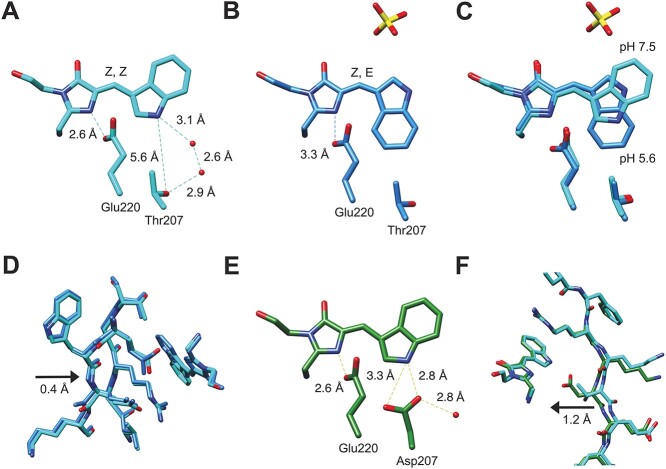
NeonCyan0.95 and NeonCyan1-T207D chromophore configuration and interactions. (**A**) The NeonCyan0.95 structure at pH 7.5 reveals a water-bridged hydrogen bond network between the indole nitrogen and the hydroxyl group of Thr207. Distances are averages calculated from all monomers in the asymmetric unit. (**B**) The chromophore of NeonCyan0.95 at pH 5.6 is the *Z*,*E* stereoisomer and ‘flipped’ relative to the *Z*,*Z* stereoisomer at pH 7.5. The pH 5.6 structure also revealed a sulfate ion bound adjacent to the chromophore. (**C)** A superimposition of the chromophores and adjacent residues of NeonCyan0.95 at pH 7.5 and 5.6. (**D**) Superimposition of NeonCyan0.95 at pH 7.5 and 5.6 illustrates a partial deformation of β-strands 10 and 11. (**E**) The NeonCyan1-T207D structure reveals that the water-bridged hydrogen bond network in NeonCyan0.95 has been displaced by the side chain of Asp207, which now forms a direct hydrogen bond between the carboxylate group and the indole nitrogen. (**F**) The superimposed structures of NeonCyan0.95 and NeonCyan1-T207D revealed a substantial displacement of the backbone in the vicinity of residue 207 on β-strand 10.

The NeonCyan0.95 structure also reveals interesting features of the chromophore microenvironment. For example, there are two well-defined hydrogen bonds between nitrogen atoms in the chromophore and amino acid side chains. One of these is between a nitrogen atom of the 4-imidazolone ring and the side chain of Glu220, and the other is between the nitrogen atom of the indole ring and a water molecule that is part of a hydrogen bond network that also contains the side chain hydroxyl of Thr207 (Fig. 4A and Supplementary Fig. 2C). In addition, one face of the chromophore is closely packed with ionizable residues with side chains potentially bearing positive charges (His72, Lys153, Arg205) and negative charges (Glu45 and Glu220) (Supplementary Fig. 2D). The close proximity of these ionizable residues is likely to impact the spectroscopic properties of the chromophore and may contribute to the modest red-shift of mNeonGreen relative to EGFP, and of NeonCyan relative to avGFP-derived CFPs. One final observation is that the bond angle of the methylene bridge (that is, the angle between the two C-C bonds attached to the Cβ of the chromophore-forming tryptophan) is particularly open (132°) compared with other CFPs such as Cerulean (124°) and mTurquoise2 (122°) (Goedhart *et al.* 2012; Gotthard *et al.* 2017). This larger bond angle is conserved in NeonCyan1-T207D (131°) but further exacerbated in the acidic pH structure (144°).

### X-ray crystal structure of NeonCyan0.95 at acidic pH

In addition to obtaining the structure of NeonCyan0.95 at physiological pH, we also obtained the structure of NeonCyan0.95 at an acidic pH (estimated to be ~5.6) which is below the apparent p*K*_a_ of NeonCyan1 (6.4, Table I). The most notable differences between two structures are the different configurations of the chromophore, which is in a *Z*,*Z* configuration at physiological pH and *Z*,*E* configuration at acidic pH, the presence of a sulfate ion bound in close proximity to the chromophore at acidic pH, and a partial deformation of β-strands 10 and 11 (Fig. 4B–D). Absorbance spectra of the NeonCyan1 variants in the presence and absence of sulfate ion, at pH 4 and 7.4, did not reveal any substantial effect on the absorption profile (Supplementary Fig. 4).

### Hue-shifted variants of NeonCyan1

Inspired by the X-ray crystal structure, which revealed a water-bridged interaction between Thr207 and the indole nitrogen of the chromophore (Fig. 4A), we further investigated the influence of residue 207 on the spectral properties of the chromophore. Screening of a library in which Thr207 was mutated to all other common amino acids led to the identification of two variants with drastically different spectral properties relative to NeonCyan1: Thr207Met (NeonCyan1-T207M) with blue shifted excitation and emission, and Thr207Asp (NeonCyan1-T207D) with red-shifted excitation and emission (Fig. 2B).

NeonCyan1-T207M and NeonCyan1-T207D were characterized in terms of their fundamental spectral and biophysical properties (Table I). Relative to NeonCyan1, NeonCyan1-T207M has a lower Φ (0.18 versus 0.29), but the apparent p*K*_a_ and ε are essentially unchanged. Relative to NeonCyan1, NeonCyan1-T207D has a similar Φ (0.27 versus 0.29) and an increased ε (42 mM^−1^ cm^−1^ versus 29 mM^−1^ cm^−1^). In contrast to NeonCyan1 and NeonCyan1-T207M, which both exhibit a sigmoidal dependence on pH that is characterized with a single p*K*_a_, NeonCyan1-T207D’s pH dependency is best fit as a biphasic curve with p*K*_a_ values of 5.4 and 8.0 (Fig. 2C). Based on this *in vitro* pH dependency, the fluorescence of NeonCyan1 and NeonCyan1-T207M are substantially more sensitive than NeonCyan1-T207D to pH changes in the physiologically relevant range (pH ~ 7).

To investigate the mechanism of color modifying mutations, we attempted to crystallize the NeonCyan variants under different pH conditions and ultimately obtained the X-ray crystal structure of NeonCyan1-T207D at pH 6.5. This structure revealed that the side chain of the Asp residue at position 207 had displaced the water molecules that had formed a hydrogen bond network from the indole nitrogen to the side chain of Thr207 (Fig. 4E). Accompanying this change was a displacement of the backbone in the vicinity of residue 207 with the Cα of Asp207 in NeonCyan1-T207D positioned 1.2 Å closer to the chromophore than the Cα of Thr207 in NeonCyan0.95 (Fig. 4F).

Suspecting that the different spectral properties for mNeonCyan1, NeonCyan1-T207D, and NeonCyan1-T207M might be due to differences in chromophore ionization state, we performed (TD)-DFT calculations to obtain insight into the relative energies and predicted absorbance maxima for the neutral, anionic, and various protonated states (in all cases, for the Z,Z isomers). Computed excited state energies, predicted absorbance maxima, oscillator strengths, and deprotonation energies, for both gas-phase models and PCM water-solvated models are provided in Supplementary Table III. Calculated gas-phase electrostatic potential (ESP) relative charges and highest occupied molecular orbital (HOMO) to lowest unoccupied molecular orbital (LUMO) gap energies are provided in Supplementary Table IV. Selected charges and bond lengths are provided in Supplementary Table V. Molecular ESP maps are represented in Supplementary Fig. 5, and HOMOs and LUMOs are graphically represented in Supplementary Fig. 6. The calculated absorbance maxima (Supplementary Table III) are not in good quantitative agreement with the experimental values, but this is unsurprising given that the calculations are for the isolated chromophores and do not include the protein environment. Regardless, the calculations provide some general trends that inform our understanding of the chromophore structure and spectral properties. Both the gas-phase and the PCM calculations predict that the anionic chromophore (‘Anion’ in Supplementary Figs 5, 6 and Supplementary Tables III–V), the chromophore protonated on the oxygen atom of the 4-imidazolone (Protonated O), and the chromophore protonated on the nitrogen atom of the 4-imidazolone (Protonated N12) have decreased ground state to excited state (or HOMO to LUMO) energy gaps relative to the ‘Neutral’ chromophore (Supplementary Tables III and IV). Accordingly, these calculations are consistent with these ionized structures having a red-shifted excitation and emission relative to the neutral chromophore. Calculations were also performed for the chromophore with protonation on the imidazole (Protonated N7). However, as this structure requires disruption of imidazole aromaticity, we consider its formation to be unlikely and it will not be further discussed.

### Live cell imaging with NeonCyan1 variants

**Fig. 5:**
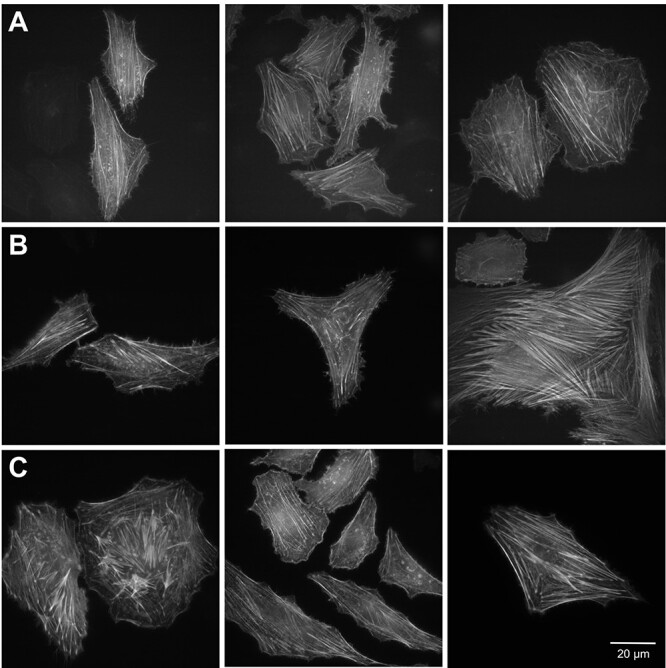
Imaging of live HeLa cells with NeonCyan1 variants targeted to actin filaments. (**A**–**C**) Representative images of HeLa cells expressing NeonCyan1 variants as fusions to the F-tractin targeting sequence. (**A**) NeonCyan1-T207M. (**B**) NeonCyan1. (**C**) NeonCyan1-T207D.

To assess the suitability of NeonCyan1 variants for live cell imaging, we expressed the corresponding genes in HeLa cells as fusions to the coding sequence of the F-tractin peptide using a modified pcDNA3.1 vector. F-tractin is derived from the N-terminal portion of rat neuronal 1,4,5-trisphosphate 3-kinase A, residues 10 through 52, and has been demonstrated to be an effective reagent for labeling of filamentous-actin (F-actin) in live cells (Belin *et al.* 2014; Brehm *et al.* 2004; Schell *et al.* 2001). HeLa cells that had been transfected with each of the pcDNA3.1-F-tractin-NeonCyan1 variant constructs were imaged using a wide-field fluorescence microscope with appropriate excitation and emission filters (Fig. 5). All three F-tractin-NeonCyan1 variant fusions enabled effective imaging of the actin cytoskeleton.

## Discussion

### Development of a new lineage of CFP variants

Our efforts to develop variants of mNeonGreen with alternative chromophore structures were a mixed success—we succeeded in creating fluorescent variants with a tryptophan-derived chromophore, but not with phenylalanine- or histidine-derived chromophores. Starting from a dimly fluorescent variant with the tryptophan-derived chromophore, we employed extensive directed evolution that ultimately led to the production of a variant designated NeonCyan1 which has nine mutations relative to mNeonGreen (Shaner *et al.* 2013). Compared with contemporary CFP variants such as mCerulean3 and mTurquoise2, NeonCyan1 has similar excitation and emission maxima and a similar extinction coefficient (ε) (Goedhart *et al.* 2012; Markwardt *et al.* 2011). However, NeonCyan1’s quantum yield (Φ) of fluorescence is substantially lower than that of mCerulean3 and mTurquoise2 (0.29 vs 0.87 and 0.93, respectively), resulting in only ~ 30% of the overall brightness (Table I). The substantially lower brightness of mNeonGreen-derived NeonCyan1 relative to the contemporary avGFP-derived CFPs is particularly disappointing because the high brightness of mNeonGreen relative to avGFP was a major motivation for this work. Specifically, our hypothesis was that the chromophore environment of mNeonGreen, which is responsible for the bright fluorescence of its Tyr-derived chromophore, would similarly enhance the fluorescence of homologs with modified chromophore structures and altered fluorescent hues. It is apparent that our results do not support this hypothesis.

### Overview of crystallographic studies of NeonCyan1 and variants

To obtain insight into the molecular basis of the empirically determined biophysical and photophysical properties of NeonCyan1 and its variants, we determined the crystal structure of NeonCyan0.95 at close to physiological pH (pH 7.5), NeonCyan0.95 at acidic pH (pH 5.6), and NeonCyan1-T207D at close to physiological pH (pH 6.5). Attempts to obtain crystal structures of NeonCyan1, NeonCyan1-T207M, and structures at other pH values were unsuccessful. Fortunately, each of the three determined crystal structures provided informative molecular insight.

In the case of NeonCyan0.95 (pH 7.5), the structure revealed the dimer–interface interactions of the protein (discussed next) as well as a water-bridged hydrogen bond network that extended from the indole nitrogen of the chromophore to the hydroxyl group of Thr207. Based on this latter observation, we suspected that mutagenesis of Thr207 of NeonCyan1 would lead to the discovery of variants with different interactions with the chromophore that may improve or otherwise modulate its photophysical properties. Pursuing this idea led to the discovery of the red-shifted NeonCyan1-T207D and blue-shifted NeonCyan1-T207M variants.

Relative to the *Z*,*Z* chromophore stereoisomer in NeonCyan0.95 at pH 7.5, the chromophore of NeonCyan0.95 at pH 5.6 was revealed to be flipped to the *Z*,*E* stereoisomer. We tentatively propose that this pH-dependent change in the chromophore configuration might be associated with reverse protonation of Glu220, which is structurally aligned with Glu222 of avGFP (Ormö *et al.* 1996). Specifically, the structure is consistent with Glu220 being protonated at physiological pH, because it engages in a strong hydrogen bond (2.65 +/− 0.02 Å; the average from the four molecules in the asymmetric unit) with the unprotonated nitrogen atom of the imidazolinone ring (Fig. 4A). At acidic pH, the distance to the nitrogen atom of the imidazolinone ring has increased to 3.34 +/− 0.16 Å, which indicates a weaker hydrogen bond and may even be consistent with Glu222 being deprotonated (Fig. 4B). Associated with this weaker interaction is an outward movement of β-strand 11, which contains Glu220, and β-strand 10 (Fig. 4D). This conformational change may help to sterically accommodate the *Z,E* stereoisomer of the chromophore.

Comparing the structure of NeonCyan0.95 (pH 7.5) with the structure of NeonCyan1-T207D (pH 6.5) revealed that the water-bridged hydrogen bond network that had extended from the indole nitrogen of the chromophore to the hydroxyl of Thr207 was displaced by the side chain of Asp207, and there was a close interaction (2.80 +/− 0.11 Å) between the indole nitrogen and the carboxyl group of Asp207. As discussed next, we tentatively propose a mechanism by which this interaction may give rise to the observed red-shift of the NeonCyan1-T207D variant.

### Structure-based insights into the oligomeric structure

The crystal structure of NeonCyan0.95 revealed that the protein forms a dimer at the high protein concentrations used for crystallization. During the original evolution of mNeonGreen from LanYFP (Shaner *et al.* 2013), the dimer interface was purposely disrupted with the Val140Arg mutation (Clavel *et al.* 2016). Generally speaking, dimeric FPs tend to be more stable and brighter than their monomeric analogs. Accordingly, when performing directed evolution for improved brightness, it is unsurprising to discover mutations that revert dimmer monomers into brighter dimers. Consistent with this expectation, four out of the nine mutations introduced in going from mNeonGreen to NeonCyan1 are within the observed dimer interface (Fig. 3). Of these mutations, we suspect that the Arg150Trp mutation likely had the greatest effect on stabilizing the dimeric structure due to the formation of a hydrophobic pocket with Phe204 and Phe225 (Supplementary Fig. 7). Overall, the localization of the mutations that accumulated during the evolution of NeonCyan1 highlights the importance of the dimer interface for stabilization of the Trp-derived chromophore structure.

### The red-shifted NeonCyan1-T207D variant

At a glance, the similarity between the red-shifted spectrum of NeonCyan1-T207D, and the spectra of WasCFP (Sarkisyan *et al.* 2012) and NowGFP (Pletnev *et al.* 2015; Sarkisyan *et al.* 2015a), ostensibly suggests that all three proteins may harbor anionic tryptophan-derived chromophores. However, inspection of the chromophore interactions in the crystal structures of NeonCyan1-T207D and NowGFP strongly suggest that this is not the case, and distinct mechanisms must be at work. The p*K*_a_ of the proton attached to the indole nitrogen of a CFP-type chromophore is ~ 12.4 (Sarkisyan *et al.* 2012), which is approximately eight units lower than the p*K*_a_ of indole itself (p*K*_a_ ~ 21) (Bordwell *et al.* 1981), due to the additional resonance stabilization associated with the extended conjugation (Fig. 1B). To promote formation of the anionic chromophore in WasCFP and NowGFP, the protein was engineered with the Val61Lys mutation to position the terminal amine of the lysine side chain 3.0 Å from the indole nitrogen (Pletnev *et al.* 2015). The approximate matching of the p*K*_a_ values between the amine side chain of lysine (typically ~10.5 and possibly higher in the context of a folded protein), and the CFP chromophore (~12.4, and possibly lower in the context of a folded protein) makes it feasible for the acid–base equilibrium to favor the co-existence of the anionic chromophore and the protonated lysine. Our computed ESP maps are consistent with the anionic form having substantial negative charge on the indole nitrogen (Supplementary Fig. 5 and Supplementary Table V).

In contrast to WasCFP and NowGFP, NeonCyan1-T207D has the terminal carboxylate of an aspartate side chain, which typically has a p*K*_a_ of ~ 4, positioned 2.80 Å from the indole nitrogen (Fig. 4E). The approximately eight units of mismatch between the p*K*_a_ of the aspartate side chain and the p*K*_a_ of the CFP chromophore are incompatible with the co-existence of the anionic chromophore and protonated aspartate. Possibly, the ionization event with a p*K*_a_ of 5.4 in the pH titration of NeonCyan1-T207D could be consistent with Asp207 existing in the deprotonated state at physiological (neutral) pH values (Table I and Fig. 2C).

How to explain the observed red-shift of NeonCyan1-T207D? While we cannot claim to have a definitive answer, our preferred explanation is that the close proximity of a positive charge to the partially negative indole nitrogen of anionic WasCFP and NowGFP (refer to resonance forms iii and iv in Fig. 1B), and the close proximity of a negative charge to the partially positive indole nitrogen of neutral NeonCyan1-T207D (refer to resonance forms i and ii in Fig. 1B) may have similar stabilizing effects on the excited state of the chromophore and cause a similar red-shift. In a hybrid orbital model of the chromophore structure, the lone pair of electrons on the deprotonated indole nitrogen are in an sp^2^-hybridized orbital that is in the plane of the aromatic ring and not part of the conjugated system. Accordingly, the resonance forms for both the neutral and anionic forms of the chromophore are very similar, and only differ in the distribution of partial charges (Fig. 1B). This approximate similarity is borne out by the similarly shaped HOMO and LUMO orbitals calculated for the anion and neutral forms (Supplementary Fig. 6). We propose that the stabilization provided by the positively charged side chain of Lys61 in WasGFP and NowCFP interacting with the partial negative charge of the anionic chromophore (as approximated by resonance form iii in Fig. 1B) is similar to the stabilization provided by the negatively charged side chain of Asp207 in NeonCyan1-T207D interacting with the partial positive charge of the neutral chromophore (as approximated by resonance form ii in Fig. 1B). We suggest that these two mechanisms of chromophore stabilization are associated with similar decreases in the energy gap between the HOMO and LUMO, resulting in similar excitation and emission peak wavelengths.

An alternative mechanism to explain the red-shift is that NeonCyan1-T207D harbors a chromophore that is protonated on either the oxygen (‘Protonated O’ in Supplementary Fig. 5) or nitrogen (‘Protonated N12’ in Supplementary Fig. 5) of the 4-imidazolone moiety. Our TD-DFT calculations predict that these chromophores would be red-shifted relative to the neutral chromophore and, as with the neutral chromophore, these structures would have partial positive character on the indole nitrogen which could be stabilized by the negatively charged side chain of Asp207. However, there is no reason to expect that the T207D mutation would depress the p*K*_a_ of the ionizable groups of the 4-imidazolone moiety sufficiently for them to be protonated at neutral pH values.

### The blue-shifted NeonCyan1-T207M variant

Relative to NeonCyan1, NeonCyan1-T207M is blue-shifted by ~20 nm. Unfortunately, we were not able to obtain a crystal structure of this protein that may have revealed changes in the chromophore configuration or interactions and suggested a possible mechanism for the change in fluorescence spectral profile. Accordingly, we turn to alternative lines of evidence and reasoning to arrive at a possible mechanistic explanation. Our TD-DFT calculations of the various possible ionization states of the tryptophan-derived chromophore suggested that the anionic or feasible protonated states would be red-shifted relative to the neutral form (Supplementary Table III). This consistent red-shift suggests that non-neutral ionization states are unlikely to be a satisfactory explanation for the observed blue-shift in the excitation and emission profile.

How to explain the blue-shift of the NeonCyan1-T207M variant? One possible explanation is that the presence of the bulky and apolar methionine residue has induced the chromophore to undergo a flip to the *Z*,*E* stereoisomer. The *Z*,*E* configuration has consistently been associated with a blue-shifted spectral profile for the neutral chromophore (Gotthard *et al.* 2017; Liu *et al.* 2017; Malo *et al.* 2007). Furthermore, the *Z*,*E* chromophore stereoisomer is a commonly observed feature in CFPs with Xaa-Trp-Gly-derived chromophores at lower pH. As examples, this configuration has been reported for Cerulean at pH 5.0 (Malo *et al.* 2007), SCFP3A at pH 4.5 (Gotthard *et al.* 2017), CyPet at pH < 7.0 (Liu *et al.* 2017), and NowGFP at pH 4.8 (20% *Z*,*E* and 80% *Z,Z*) (Pletnev *et al.* 2015). Similarly, the crystal structure of NeonCyan0.95 at pH 5.6 revealed that the chromophore is in the *Z*,*E* configuration (Fig. 4B). This observation supports the conclusion that the NeonCyan1 structure can accommodate the *Z*,*E* stereoisomer. Another second explanation is that the chromophore remains as the *Z,Z* stereoisomer and the bulky and apolar methionine residue has simply displaced the water molecules in the water-bridged hydrogen bond network between the indole nitrogen of the chromophore and the hydroxyl group of Thr207 (refer to Fig. 4A). In this situation, the indole nitrogen might not be hydrogen bonded to any atom (either water- or protein-derived), leading to a loss of excited state stabilization and a corresponding blue-shift in the fluorescence profile.

### Quantum yields and the chromophore conformation

Based on inspection of the crystal structures, we hypothesize that the low quantum yield of NeonCyan1 and NeonCyan1-T207D relative to the best avGFP-derived CFPs is related to the non-coplanarity between the imidazolinone and indole rings of the chromophore. The first and second dihedral angles Φ_1_ and Φ_2_ of the chromophore around the methylene bridge are, respectively, 15.2° +/− 0.6° and − 9.9° +/− 0.7° for the four molecules in the crystal structure of NeonCyan0.95 at pH 7.5, and 17.0° +/− 2.8° and − 7.8° +/− 3.1° for the four molecules in the crystal structure of NeonCyan1-T207D. In contrast, the average values of Φ_1_ and Φ_2_ are 6.3° +/− 0.7° and − 5.5° +/− 1.6° for the near-physiological pH structures of four of the most used avGFP-derived CFPs: ECFP (PDB ID: 2WSN) (Lelimousin *et al.* 2009), Cerulean (PDB ID: 5OXC) (Gotthard *et al.* 2017), mTurquoise (PDB ID: 2YE0) (Goedhart *et al.* 2012), and mTurquoise2 (PDB ID: 3ZTF) (Goedhart *et al.* 2012). In these avGFP-derived CFPs, the absolute value of Φ_1_ and Φ_2_ are similar, and so their opposite signs result in the two rings being roughly coplanar. In both NeonCyan0.95 and NeonCyan1-T207D, the first dihedral angle Φ_1_ is substantially larger than its value in the avGFP-derived CFPs, and cannot be compensated by the second dihedral angle Φ_2_, making the two rings non-coplanar. This distortion is essentially due to the sandwiching of the indole ring between Pro55, Phe155, and Arg195. The same sandwiching in the highly fluorescent mNeonGreen is compatible with the rings being coplanar, presumably due to the smaller size of the phenolate compared with the indole ring. The non-coplanar chromophore conformation in the NeonCyan series explains their moderate QY. It is possible that a higher quantum yield and enhanced brightness could be achieved by further protein engineering, but this would require untwisting the indole ring while maintaining a rigid surrounding environment.

### Summary and prospects for the NeonCyan lineage

In conclusion, we have developed and thoroughly characterized three spectrally distinct variants of mNeonGreen that all share a Gly-Trp-Gly-derived chromophore. This work provides insight into the interactions that influence the properties of tryptophan-derived chromophores, and reveal that it is indeed possible to modify the covalent structure of the mNeonGreen chromophore in order to produce hue-shifted variants. While these variants promisingly appear to retain mNeonGreen’s usefulness for live cell imaging experiments, they unfortunately do not retain the desirable high fluorescent brightness of mNeonGreen. At this point, we recommend that researchers use other contemporary CFPs, such as mCerulean3 and mTurquoise2, rather than NeonCyan1 variants, for fluorescent imaging applications (Goedhart *et al.* 2012; Markwardt *et al.* 2011). However, we remain optimistic that the combination of structure-guided protein engineering and directed protein evolution may ultimately produce improved variants of NeonCyan1 that are bright, monomeric, and photostable, and accordingly of high utility for biological imaging applications.

## Authors’ Contributions

L.Z., R.E.C., A.B., and A.R. designed the experiments. L.Z., H.B., and R.E.C. performed and interpreted the protein engineering and characterization experiments. D.C., K.D., H.D., J.D., and A.R. performed and interpreted protein crystallography experiments. R.J. and A.B. performed and interpreted computational experiments. R.E.C., A.R., and A.B. supervised the research. L.Z., A.B., A.R., and R.E.C. wrote the manuscript.

## Supplementary Material

NeonCyan_Supplementary_Information_21_11_22_revised_gzac004
