# Supplementary material for: Cyan fluorescent proteins derived from mNeonGreen
Source: Protein Eng Des Sel. 2022 May 9;35:gzac004. doi: 10.1093/protein/gzac004 (PMC9083105; doi:10.1093/protein/gzac004)
Supplement: NeonCyan_Supplementary_Information_21_11_22_revised_gzac004 [file neoncyan_supplementary_information_21_11_22_revised_gzac004.docx]

# Supplementary Information


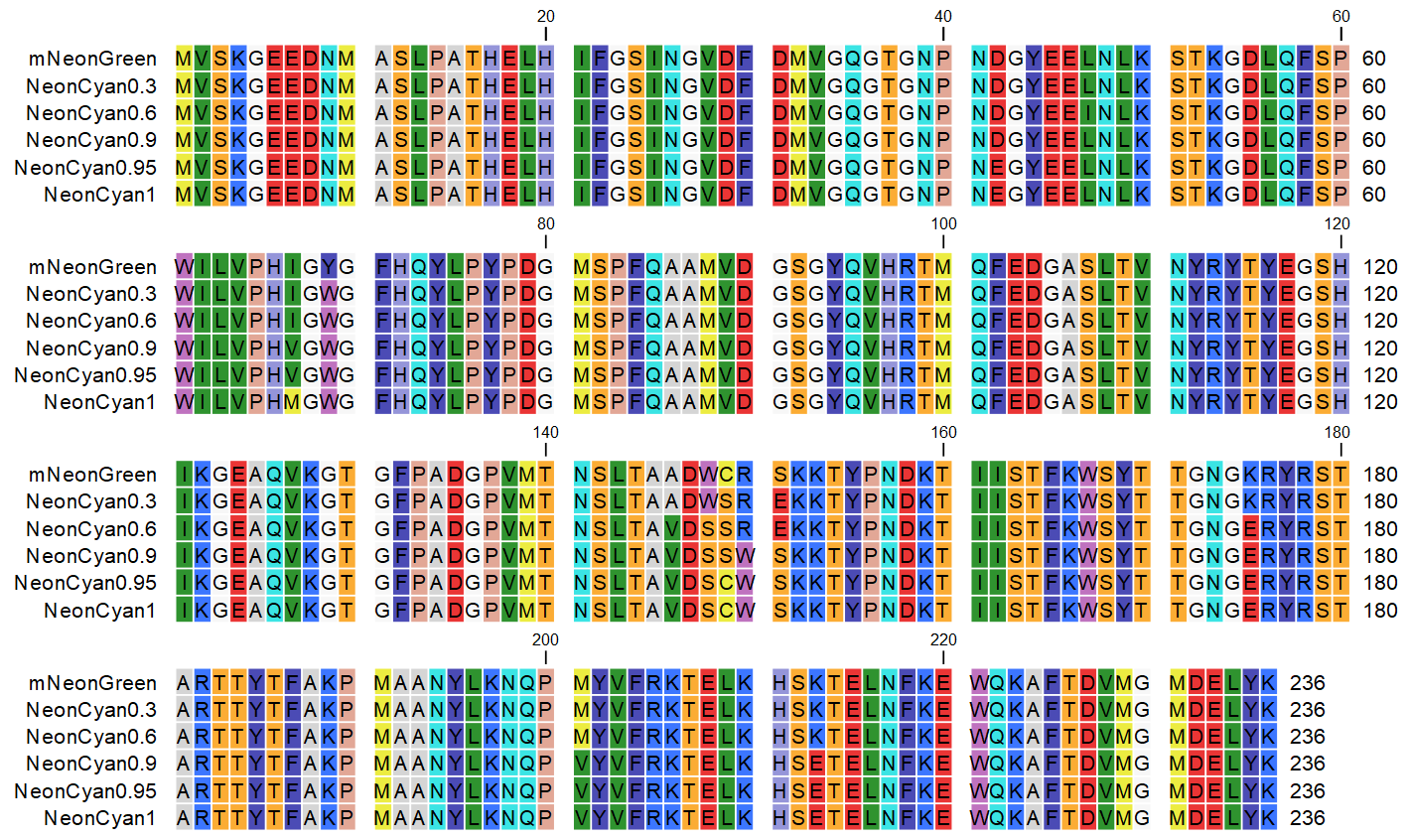
**Supplementary Fig. 1.** Sequence alignment of mNeonGreen, NeonCyan1, and various intermediates.


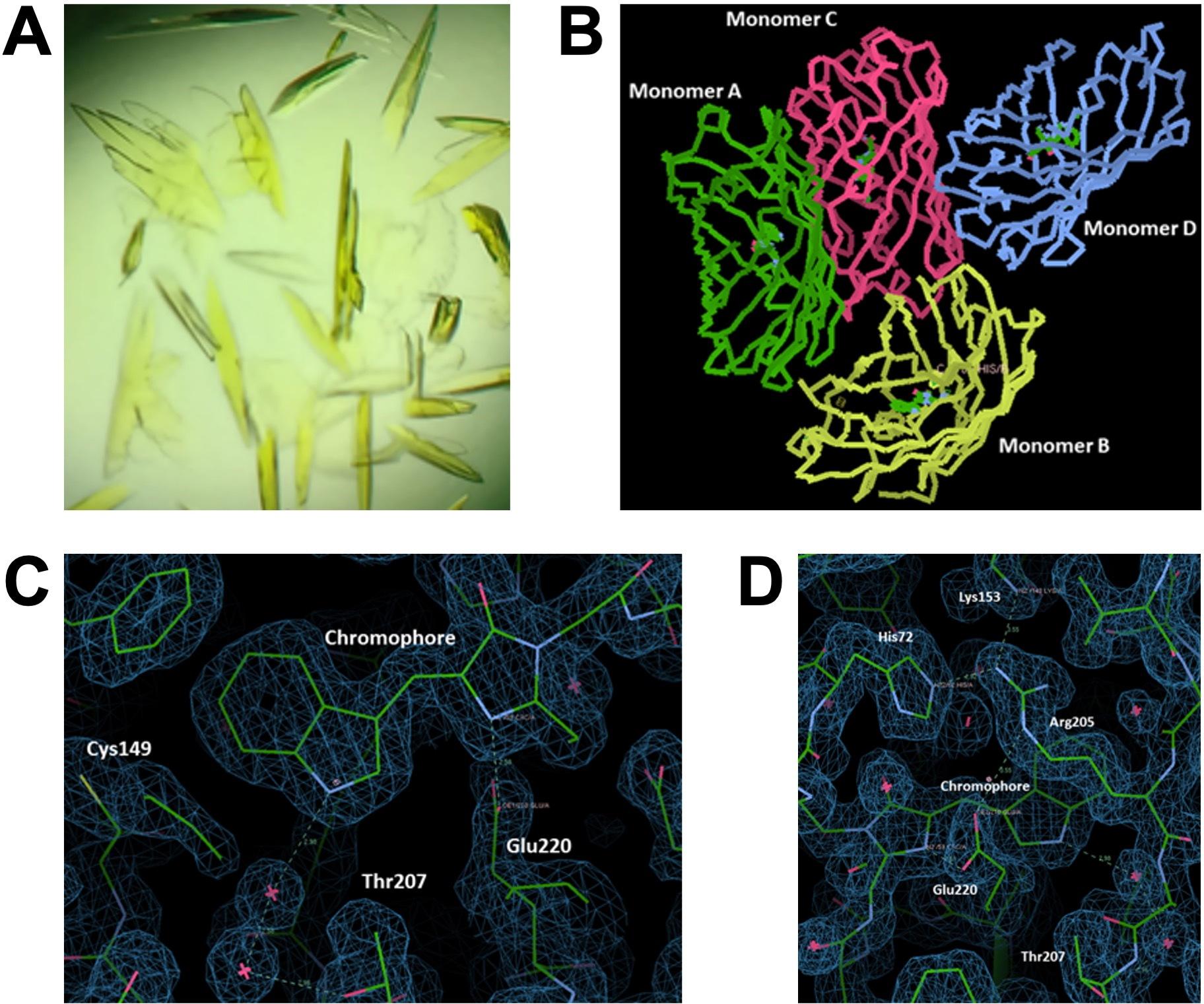

**Supplementary Fig. 2.** Overview of the NeonCyan0.95 (pH 7.5) crystal structure. (**A**) Needle-shaped crystals of NeonCyan0.95 formed within one week in a solution that contained 0.1 M HEPES pH 7.5 and 18% w/v PEG 8000. (**B**) Content of the asymmetric unit of the NeonCyan0.95 crystal lattice. (**C**-**D**) Representative electron density and key residues in the chromophore environment. Electron density superimposed on the protein structure at the chromophore location (contoured at the 1.5σ level). In (**C**), two hydrogen bonds between the chromophore and a water molecule and Glu220 are represented. In (**D**), several charged residues in the immediate vicinity of the chromophore are labeled.


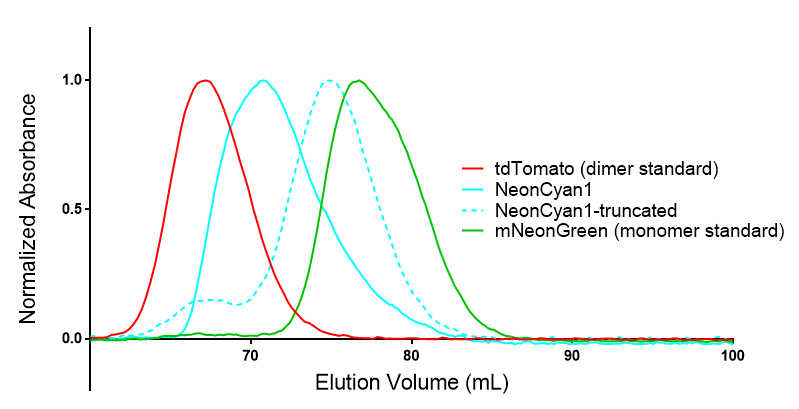

**Supplementary Fig. 3.** FPLC trace of NeonCyan1 and NeonCyan1-truncated. Size exclusion chromatography of tdTomato (dimer standard), NeonCyan1, NeonCyan1-truncated, and mNeonGreen (monomer standard). Each sample was run individually under identical conditions with absorbance-based detection at 555 nm, 430 nm, 430 nm, and 505 nm, respectively. Removal of the structured C-terminal ‘MDELYK’ peptide sequence in NeonCyan1-truncated induced a substantial shift from dimeric to monomeric character.


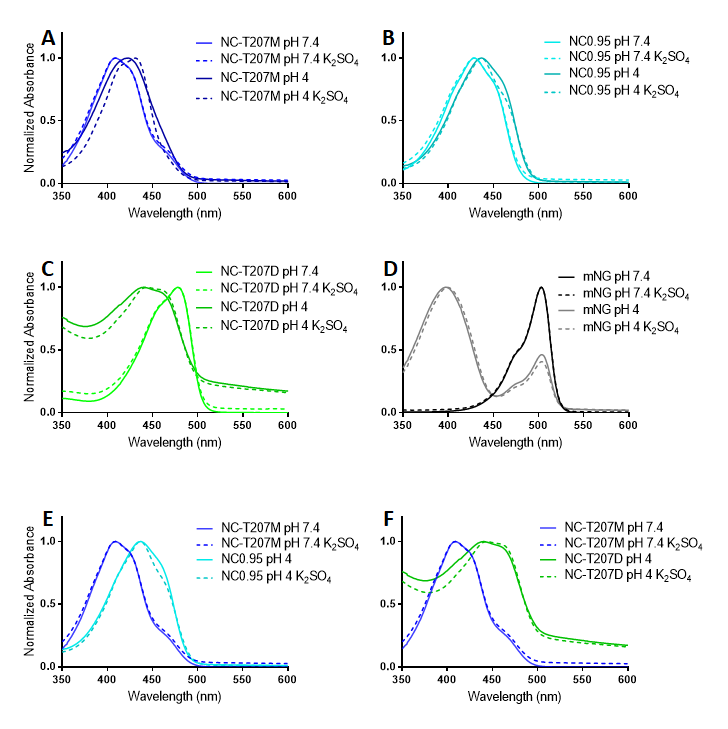

**Supplementary Fig. 4.** (**A-F**) Absorbance spectra of NeonCyan1 variants and mNeonGreen with and without 10 mM K_2_SO_4_.


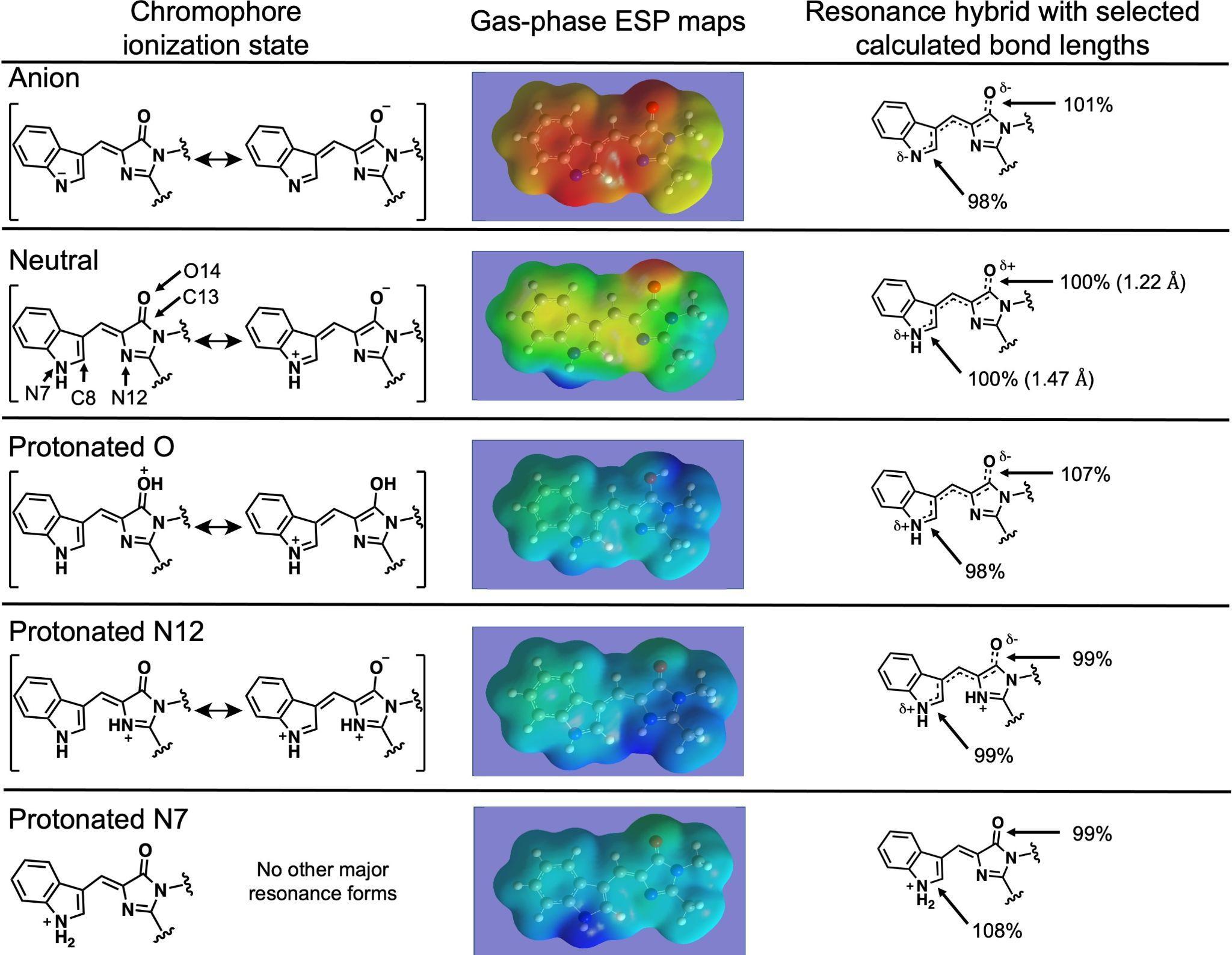
**Supplementary Fig. 5**. Calculated (ωB97xD/cc-pVDZ) electrostatic potential (ESP) maps and selected bond lengths for various possible ionization states in the gas-phase. Analogous calculations using Polarizable Continuum Model (PCM) gave qualitatively identical ESP maps. Relative charges for ESP maps are provided in **Supplementary Table 4** and selected natural charges and bond lengths are summarized in **Supplementary Table 5** for the atoms numbered on the representation of the Neutral form. Bond lengths are presented as percentages relative to the Neutral form. For the Anion and Protonated O forms, the longer C-O and shorter C-N bond lengths suggest that the relative contribution of the enol(ate)-containing resonance form has increased relative to the Neutral form. For the Protonated N12 form, the relative contributions of the two resonance forms is similar to that of the Neutral form. For the Protonated N7 form, the shorter C-O and longer C-N bond lengths suggest the resonance hybrid more closely resembles the carbonyl-containing resonance form.


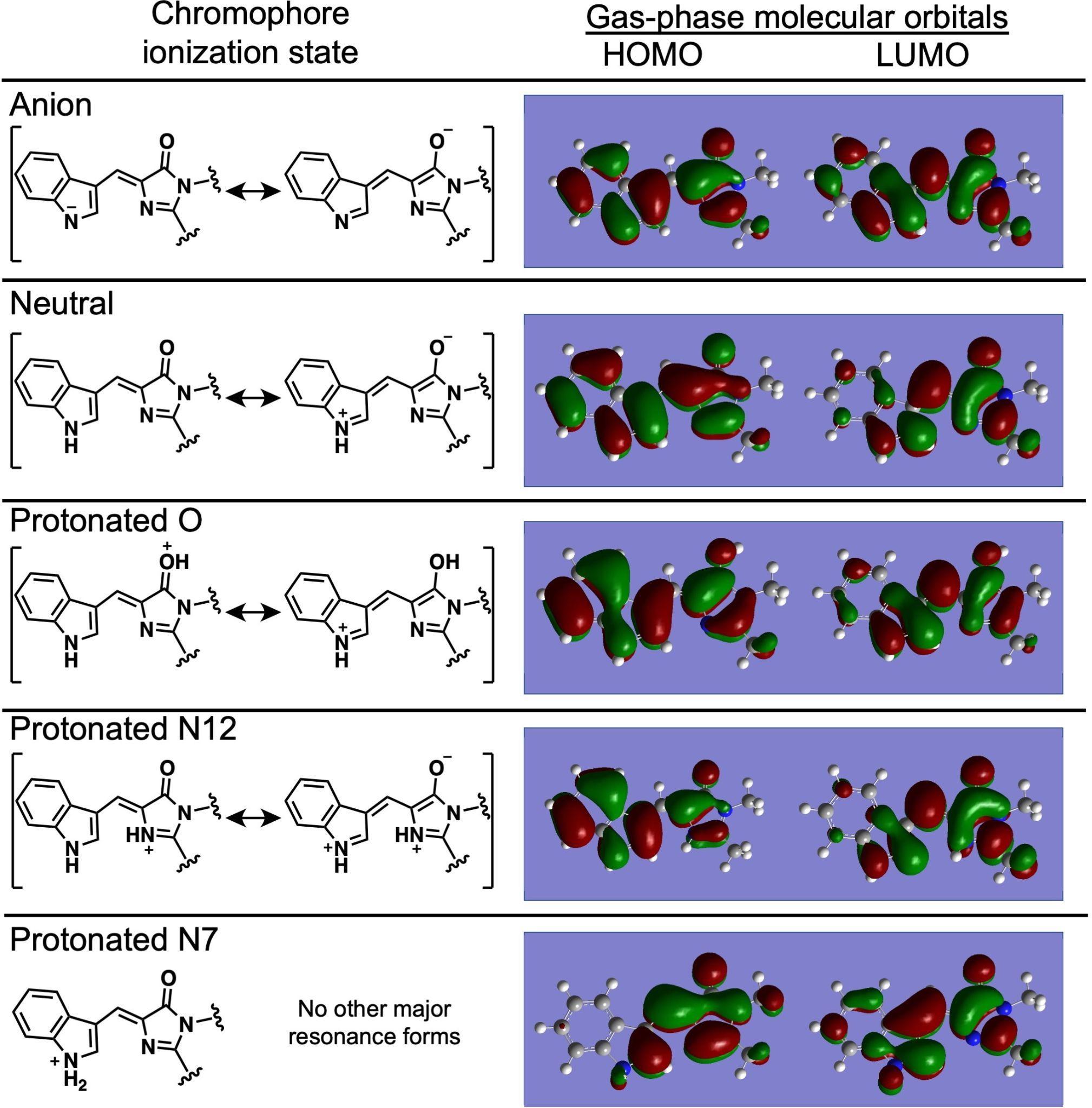


**Supplementary Fig. 6**. Calculated (ωB97xD/cc-pVDZ) HOMOs and LUMOs for various possible ionization states in the gas-phase. Calculated HOMO and LUMO energies are provided in **Supplementary Table 4**.


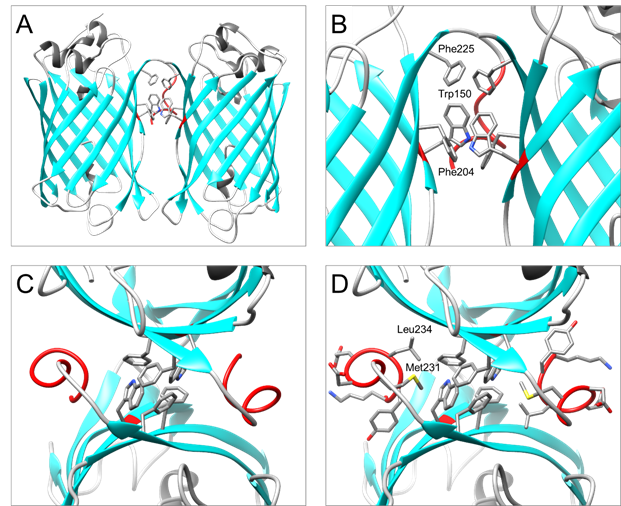


# Supplementary Fig. 7. Hydrophobic pocket of NeonCyan0.95 dimer interface. The Arg150Trp mutation introduced in NeonCyan0.7 resulted in the formation of a hydrophobic pocket within the dimer interface, as revealed in the crystal structure. (A) Hydrophobic pocket of the dimer interface of NeonCyan0.95. (B) zoomed in view of pocket residues: Trp140, Phe204, and Phe225, symmetrically interacting with the same 3 residues across the dimer interface. (C) Top-view of pocket with C-terminal ‘MDELYK’ ribbon backbone colored red. (D) Top-view of pocket with both C-terminal ‘MDELYK’ residues shown. Two hydrophobic residues of the structured C-terminal ‘MDELYK’, Met231 and Leu234, are pointed towards the hydrophobic pocket.

# Supplementary Table 1. Oligonucleotide and synthetic gene fragments used in this work.

| Name | Sequence (5’ to 3’) |
| --- | --- |
| MVSK Xho1 Fwd | TATATCTCGAGGATGGTGAGCAAGGGCGAGGAG |
| MDELYK H3 Rv | ATATAAAGCTTTTACTTGTACAGCTCGTCCATGCC |
| NC XWG QC | CCCTGGATTCTGGTCCCTCATGTCNNKTGGGGCTTCCATCAGTACCTG |
| NC XFG QC | CCCTGGATTCTGGTCCCTCATGTCNNKTTTGGCTTCCATCAGTACCTG |
| NC XHG QC | CCCTGGATTCTGGTCCCTCATGTCNNKCATGGCTTCCATCAGTACCTG |
| NC XXWG QC | CCCCTGGATTCTGGTCCCTCATNNKNNKGGGTGGGGCTTCCATCAGTACCTGCCC |
| NC 0.7 E151X QC | GCTGTGGACTCGTCCTGGNNKAAGAAGACTTACCCCAACGACAAAACC |
| NC 0.8 S149X_S151X QC | CTGACCGCTGTGGACTCGNNKTGGNNKAAGAAGACTTACCCCAACGACAAAAC |
| NC Arg150X QC | CCGCTGTGGACTCGTGCNNKAGTAAGAAGACTTACCCCAACGACAAAACC |
| pcDNA MVSK Vec Fwd | ATGGTGAGCAAGGGCTCGG |
| pcDNA FT GA Rv | GGTGGCGGCACGCTAGCC |
| F-tractin gBlock | GCTAGCGTGCCGCCACCATGGCTAGACCCCGAGGTGCCGGCCCTTGTAGTCCTGGTCTGGAGCGCGCTCCTCGACGCTCAGTAGGTGAACTGAGGTTGTTGTTCGAGGCCAGATGTGCTGCTGTGGCCGCTGCAGCAGCTGCCGGAGGGTTGGCTCTGCCGGTAGCCACCGGCGGGAGCGGGGGCAGCTCGAGGATGGTGAGCAAGGGCTCGG |
| NC Thr207X QC | GCCGGTGTACGTGTTCCGTAAGNNKGAGCTCAAGCACTCCGAGACC |

#

# Supplementary Table 2. Data reduction and structure refinement statistics.

|  | **NeonCyan0.95**  **(physiological pH)** | **NeonCyan0.95**  **(acidic pH)** | **NeonCyan1-T207D** |
| --- | --- | --- | --- |
| **PDB ID** | 7Z7O | 7Z7P | 7Z7Q |
| **Data collection** | | | |
| **Beamline** | MASSIF-1 (ESRF) | BL13-XALOC (ALBA) | BL13-XALOC (ALBA) |
| **Wavelength** | 0.966 | 0.979 | 0.979 |
| **Resolution range** | 63.7 - 1.51 (1.64 - 1.51)* | 48.9 - 1.95 (2.0 - 1.95) | 40.1 - 1.60 (1.66 - 1.60) |
| **Space group** | P 1 | P 1 | P 1 |
| **Unit cell a, b, c (Å)** | 49.85 60.66 67.07 | 66.15 72.37 117.36 | 50.06 57.98 71.45 |
| **Unit cell α, β, γ (°)** | 89.9 71.8 90.0 | 90.2 90.0 90.1 | 68.6 89.9 89.6 |
| **Total reflections** | 127 042 (6 974) | 532 256 (40 934) | 143 284 (21 273) |
| **Unique reflections** | 78 450 (3923) | 152 394 (15 160) | 85 638 (8 266) |
| **Multiplicity** | 1.6 (1.8) | 3.5 (2.7) | 1.7 (2.6) |
| **Completeness (%)** | 84.7 (51.4) | 96.0 (95.7) | 86.7 (83.6) |
| **Mean I/σ(I)** | 6.4 (1.4) | 9.7 (1.7) | 8.8 (2.4) |
| **Wilson B-factor (Å^2^)** | 26.4 | 37.5 | 16.6 |
| **R-meas (%)** | 4.6 (57.7) | 7.8 (86.8) | 10.9 (75.4) |
| **CC_1/2_** | 0.999 (0.840) | 0.997 (0.706) | 0.993 (0.703) |
| **Structure refinement** | | | |
| **Resolution range** | 63.7 - 1.51 (1.55 - 1.51)* | 48.9 - 1.95 (2.0 - 1.95) | 40.1 - 1.60 (1.64 - 1.60) |
| **Reflections used in refinement** | 112 268 (8 599) | 145 259 (10 701) | 81 404 (5 620) |
| **Reflections used for R-free** | 3 938 (19) | 7 140 (536) | 4 327 (303) |
| **R-work (%)** | 16.3 (23.2) | 20.9 (31.4) | 22.3 (45.8) |
| **R-free (%)** | 18.6 (25.0) | 25.4 (33.2) | 25.5 (52.2) |
| **Number of molecules in a.u.** | 4 | 8 | 4 |
| **Protein residues** | 887 | 1 775 | 865 |
| **Number of non-hydrogen atoms** | 7 596 | 14 907 | 7 320 |
| **protein** | 7 022 | 14 203 | 6 891 |
| **chromophore** | 84 | 168 | 84 |
| **solvent** | 490 | 496 | 345 |
| **ligand** | 0 | 40 | 0 |
| **Average B-factor (all atoms)** | 31.0 | 42.7 | 16.9 |
| **protein** | 30.7 | 42.8 | 16.7 |
| **chromophore** | 26.6 | 36.5 | 13.4 |
| **solvent** | 36.9 | 41.4 | 20.9 |
| **ligand** | - | 47.6 | - |
| **RMSD(bonds)** | 0.010 | 0.009 | 0.010 |
| **RMSD(angles)** | 1.68 | 1.71 | 1.72 |
| **Ramachandran favored (%)** | 98.1 | 96.6 | 98.8 |
| **Ramachandran allowed (%)** | 2.0 | 3.3 | 1.2 |
| **Ramachandran outliers (%)** | 0.0 | 0.1 | 0.0 |
| **Rotamer outliers (%)** | 1.5 | 1.2 | 0.8 |
| **Clashscore** | 4.2 | 4.5 | 3.3 |
| *Statistics for the highest-resolution shell are shown in parentheses. | | | |

**Supplementary Table 3**. Calculated (ωB97xD/cc-pVDZ) energy differences between the ground state (S_0_) and first excited state (S_1_), predicted peak absorbance wavelengths, and deprotonation energies of the neutral, anionic, and various protonated forms of the isolated chromophore in both gas-phase and PCM water. Deprotonation energies are calculated using the difference in DFT energies.

|  | Form | S_0_ to S_1_ energy difference (eV) | Wavelength (nm) | Oscillator Strengths | Deprotonation energy (kj/mol) |
| --- | --- | --- | --- | --- | --- |
| PCM | Anion | 3.22 | 385 | 1.0677 | 1313 |
|  | Neutral | 3.56 | 348 | 0.8173 |  |
|  | Protonated O | 3.21 | 387 | 0.9013 | 1126 |
|  | Protonated N12 | 3.37 | 368 | 0.8727 | 1182 |
|  | Protonated N7 | 3.60 | 344 | 0.4076 | 1060 |
| Gas-  Phase | Anion | 3.49 | 355 | 0.9898 | 1470 |
|  | Neutral | 3.71 | 334 | 0.7082 |  |
|  | Protonated O | 3.22 | 385 | 0.6860 | 985 |
|  | Protonated N12 | 3.27 | 379 | 0.6525 | 1015 |
|  | Protonated N7 | 3.47 | 357 | 0.0002 | 893 |

**Supplementary Table 4**. ESP relative charges and HOMO and LUMO energy gaps at the (ωB97xD/cc-pVDZ) level of theory. ESP relative charges are the upper and lower bounds of the colour gradients in **Supplementary Figure 5**.

|  | Form | ESP relative charge | HOMO to LUMO energy gap (eV) |
| --- | --- | --- | --- |
| PCM | Anion | 0.183 | 6.61 |
|  | Neutral | 0.076 | 7.04 |
|  | Protonated O | 0.213 | 6.62 |
|  | Protonated N12 | 0.231 | 6.71 |
|  | Protonated N7 | 0.233 | 7.32 |
| Gas-Phase | Anion | 0.166 | 6.57 |
|  | Neutral | 0.062 | 7.09 |
|  | Protonated O | 0.192 | 6.47 |
|  | Protonated N12 | 0.198 | 6.40 |
|  | Protonated N7 | 0.205 | 7.13 |

**Supplementary Table 5**. Selected natural charges and bond lengths calculated with natural population analysis (NPA) at the (ωB97xD/cc-pVDZ) level of theory. Atom labels are as represented on the Neutral form structure in **Supplementary Figure 5**.

| Form | N12 Natural Charge | N7 Natural Charge | O14 Natural Charge | C13-O14 Bond length (Å) | N7 - C8 Bond length (Å) |
| --- | --- | --- | --- | --- | --- |
| Anion | -0.542 | -0.615 | -0.720 | 1.233 | 1.330 |
| Neutral | -0.543 | -0.573 | -0.669 | 1.222 | 1.359 |
| Protonated O | -0.480 | -0.538 | -0.648 | 1.310 | 1.336 |
| Protonated N12 | -0.495 | -0.551 | -0.614 | 1.211 | 1.348 |
| Protonated N7 | -0.541 | -0.627 | -0.619 | 1.213 | 1.472 |

# 
